# Impact of local COVID-19 incidence on health care personnel risk perception and social activity

**DOI:** 10.1016/j.ajic.2025.10.013

**Published:** 2025-10-25

**Authors:** Holly Shoemaker, Haojia Li, Yue Zhang, Jeanmarie Mayer, Vanessa Stevens, Angela Fagerlin, Michael Rubin, Candace Haroldsen, Morgan M. Millar, Andrew T. Pavia, Per H. Gesteland, Lindsay T. Keegan, Matthew Samore

**Affiliations:** a Department of Population Health Sciences, Spencer Fox Eccles School of Medicine, University of Utah, Salt Lake City, UT; b IDEAS Center of Innovation, Veterans Affairs Salt Lake City Health Care System, Salt Lake City, UT; c Division of Epidemiology, Spencer Fox Eccles School of Medicine, University of Utah, Salt Lake City, UT; d Department of Veterans Affairs, VA Salt Lake City Healthcare System, Salt Lake City, UT; e Division of Pediatric Infectious Diseases, Spencer Fox Eccles School of Medicine, University of Utah, Salt Lake City, UT; f Division of Pediatric Hospital Medicine, Spencer Fox Eccles School of Medicine, University of Utah, Salt Lake City, UT

**Keywords:** Longitudinal survey, SARS-Cov-2, Risk communication, Pandemic workforce, Prospective cohort, Epidemiology

## Abstract

**Background:**

Risk perceptions and social activities shaped SARS-CoV-2 transmission. However, most related studies are cross-sectional, often neglecting correlated outcomes and dropout bias. We assessed relationships between local COVID-19 incidence levels and health care personnel (HCP) risk perceptions and social activity count.

**Methods:**

We conducted a prospective cohort study using monthly surveys (December 2021-May 2022) at an academic health care system (n = 1,590 HCP). COVID-19 incidence was categorized into 4 levels, from low to high. Five risk perception measures (scaled 1–4) and activity count were modeled simultaneously using a Bayesian joint model to estimate the impact of incidence on risk perception and behavior.

**Results:**

COVID-19 incidence was associated with various risk perception measures and activity count. Strongest risk perception associations were *perceived risk of infection* and *risk while unmasked indoors in public*, with score increases of 0.60 (95% credible interval [CI]: 0.51, 0.69) and 0.62 (95% CI: 0.52, 0.71), respectively, during high incidence. Individuals reported 0.68 (95% CI: −0.88, −0.47) fewer social activities during high incidence levels.

**Conclusions:**

During the Omicron surge, HCP adjusted their risk perceptions and behaviors in response to changing risk and placed high value on the protective effects of masking. These findings can inform communication strategies during future outbreaks.

## BACKGROUND

During the COVID-19 pandemic, individual decisions—such as attending social gatherings, wearing masks, or getting vaccinated—influenced community transmission of SARS-CoV-2.^1,2^ Studies have shown that adherence to preventive health behaviors was associated with higher risk perception^3,4^ and changed over the course of the pandemic.^4^ A common limitation of many studies on COVID-19 risk perception is their reliance on cross-sectional data.^5–10^ However, actual and perceived risk changed, often rapidly, as the pandemic evolved. Moreover, risk perception and willingness to alter behavior may have been related to health literacy and personal exposure to the consequences of infection. Therefore, we investigated risk perception and behaviors among health care personnel (HCP), who had a unique perspective on the pandemic.

HCP often faced unique challenges during the pandemic,^11^ and generally perceived the risks during the pandemic as higher than did the broader population.^5,6^ Understanding how HCP perceived risks and adapted their behavior over time, particularly in response to disease surges, is crucial for informing future public health responses. To address gaps in our understanding of HCP risk perception and behavior, we conducted a longitudinal survey of HCP practicing within the University of Utah health care system. Our survey included the spike in cases from the Omicron surge during the winter of 2022. This provided a natural experiment with the ability to observe perceptions and behaviors during substantial changes in external risk.

Our objective was to assess the association between local community COVID-19 levels and HCP risk perceptions and behaviors over time during the Omicron surge. We captured the complexity of risk perception by simultaneously assessing multiple aspects of risk perception alongside reported social activity. This approach uses robust methods to gain insight into how HCP respond to changing external risks in a real-world environment.

## METHODS

### Setting and participants

The study was a prospective cohort study using monthly surveys from December 2021 to May 2022. Eligible participants were HCP in a variety of roles working in patient care areas in an academic health care system, as described elsewhere.^12^ Recruitment occurred through email, flyers, and clinical livestreams, based on a list from human resources of individuals with clinical job codes. Surveys were sent via email with follow-up reminders sent 30 days later. Participation was voluntary and without compensation. Informed consent was obtained from all participants prior to the administration of the survey. The study was approved by the Institutional Review Board of the University of Utah (IRB 00145823).

Use of nonpharmaceutical interventions in Utah such as masking was variable during this time. Masking was required of all individuals in the health care facilities where the study was carried out. A statewide mask mandate for the public ended on April 10, 2021, before the study period. However, community mask mandates continued in Salt Lake County until January 2022.

### Exposure

The primary exposure in this study was COVID-19 community incidence levels, similar to those defined by the COVID-19 community transmission levels published by the centers for disease control and prevention early in the pandemic^13^ for the Salt Lake health district during the study time period. There was little variation in percent positivity during the study time period compared to new cases per 100,000 persons. Therefore, we elected to define incidence levels using the new cases per 100,000 persons alone. Incidence levels were categorized as low when between 0 to 9.99 cases per 100,000, moderate between 10 to 49.99, substantial between 50 to 99.99, or high when ≥ 100 cases per 100,000 persons. This measure of new cases per 100,000 persons does not include antigen testing, as rapid antigen tests were available to the public but not reportable in Utah during the time of the study.

### Outcomes

#### Risk perception

Risk perception was assessed through 5 questions (Supplementary Table S1). These questions assessed different aspects of risk perception: (1) perceived risk of COVID-19 in the next month, (2) perceived risk while unmasked indoors in public, (3) perceived risk while masked indoors in public while others are unmasked, (4) perception of the seriousness of impact of COVID-19 on the HCP’s health, and (5) to what degree the HCP felt risk of COVID-19 in the next month depended on their use of precautions and actions outside of work. The first 3 questions used a 4-point scale ranging from No risk (1), Low risk (2), Moderate risk (3), and High risk (4). Questions 4 and 5 used a 5-point scale: Not applicable, I am not at risk of getting COVID-19 (1), No impact/Not dependent (2), Small impact/Slightly dependent (3), Moderate impact/Moderately dependent (4), and Significant impact/Highly dependent (5). To keep all questions on the same 4-point scale, the 5-point questions had the “No impact”/”Not dependent” and “Not applicable” categories combined. All risk perception variables were treated as numeric in the analysis, with higher numbers corresponding to higher risk perception.

We hypothesized that several dimensions of perceived COVID-19 risk would be associated with local incidence levels. These included: (1) perceived risk of COVID-19 in the next month; (2) perceived risk while unmasked indoors in public; (3) perceived risk while masked indoors in public while others are unmasked; and (4) the extent to which participants believed their risk in the next month depended on their personal precautions and behaviors outside of work. In contrast, we hypothesized that the perceived seriousness of COVID-19’s potential impact on an HCP’s health would not be associated with local incidence levels. In the context of the survey, “next month” refers to the month following each participant’s response. For example, if a participant completed the survey in December, next month would refer to January.

#### Behavior

Behaviors potentially associated with Covid-19 were assessed by asking: “In the past month, did you do any of the following?” (Supplementary Table S2). Activities included:

- Socialized at a home indoors with nonhousehold members,
- Socialized outdoors with nonhousehold members,
- Socialized or attended an indoor event in public (ie, concert/movies/sporting),
- Socialized or attended an outdoor event in public (ie, concert/movies/sporting),
- Attended in-person religious services,
- Gone to a store (ie, grocery, retail, etc),
- Gone to a gym or fitness center,
- Eaten indoors at a restaurant
- Traveled on an airplane.

Activities were combined into a count of activities ranging in value from 0 to 9. We hypothesized that HCP would modify their behavior and show reduced social activities during periods of increased incidence.

#### Analysis

We calculated descriptive statistics (counts and percentages) for study respondent demographic characteristics. Pearson’s correlation was used to assess the correlation between outcome variables. We utilized a Bayesian joint model^14,15^ to simultaneously evaluate the impact of COVID-19 incidence levels on all risk perception questions and the count of social activities. This strategy allows us to retain information from all risk perception questions rather than rely on a combined variable, account for the correlation between variables,^16^ and borrow statistical power across multiple outcomes.^17^ Additionally, we adjust for likelihood of dropout with covariate balancing propensity score weighting^18^ (Supplementary Fig. S1).

#### Covariates

Based on covariates that were included in other studies,^19,20^ this model was adjusted for gender, age group, work role, one or more comorbidities (compared to none listed), general health status, months since most recent vaccine, months since most recent COVID-19 infection, work location, number of people in household and school age children, and survey month. Several variables were collapsed from their original survey options for simplicity (Supplementary Table S3). The survey month was kept separate for descriptive statistics, but December and January were combined in the model due to lower sample sizes. For additional detail on how covariates were collapsed, please see Shoemaker et al.^12^

## RESULTS

### Summary statistics

**Fig. 1.**
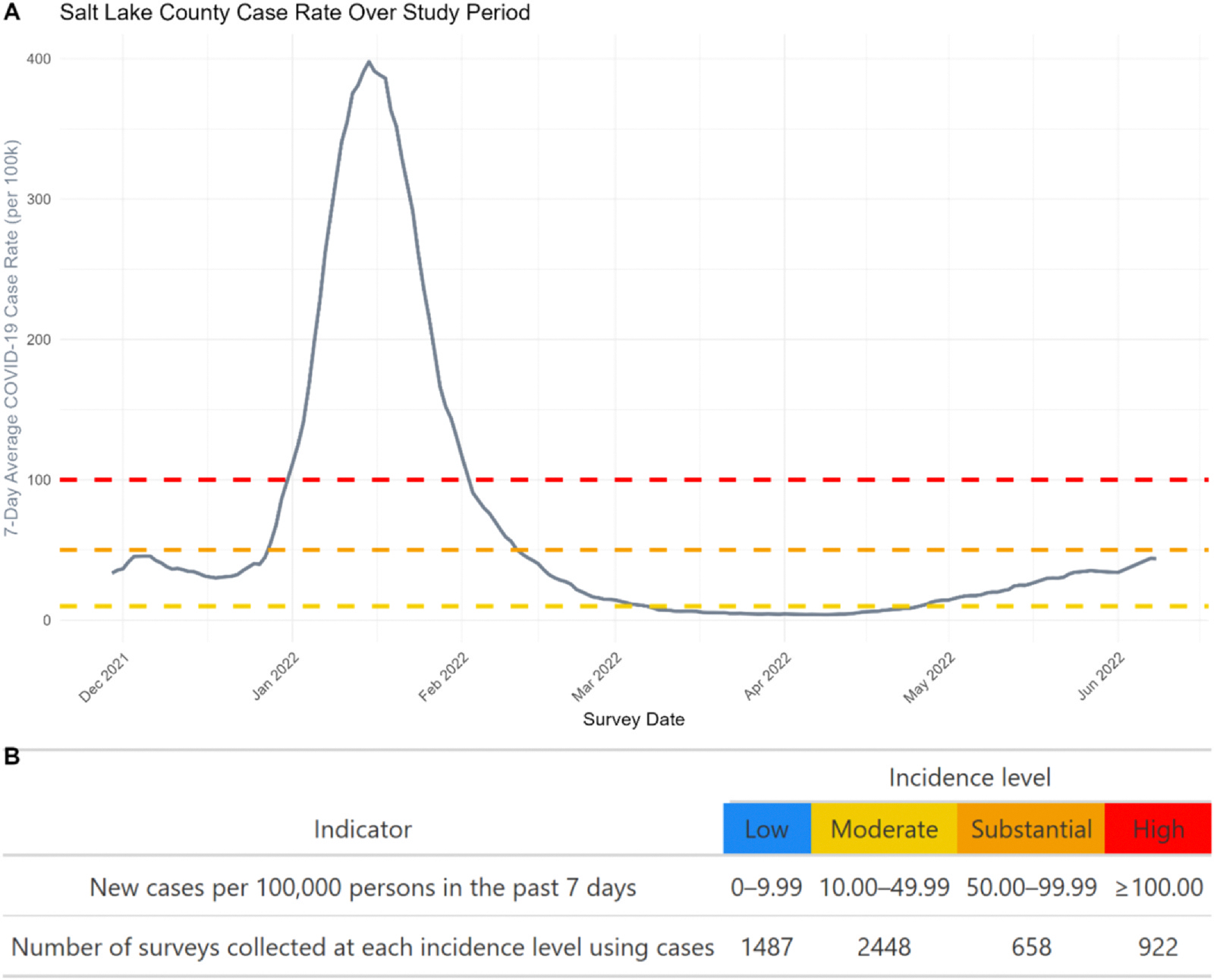
(A) Average 7-day case rate per 100,000 over the study period. Dashed lines show thresholds for incidence level based on new cases, corresponding to the colors in the table below (with red indicating high, orange indicating substantial, and yellow indicating moderate. Low begins at 0 and is not shown on the graph with a line). (B) Incidence level thresholds based on the community transmission levels originally defined by the CDC and the number of surveys which fall into each category.

Of the 10,321 HCP sent recruitment emails, a total of 1,590 HCP (15%) participated in the study by consenting and completing at least one survey. Most participants were younger than 45 years (68%, median = 35–44), and a majority were female (79%) (Table 1). Most reported excellent or very good health (73%), living with others, and no school-age children (60%). Cases during the study reached peak levels during January and February (Fig. 1).

**Fig. 2.**
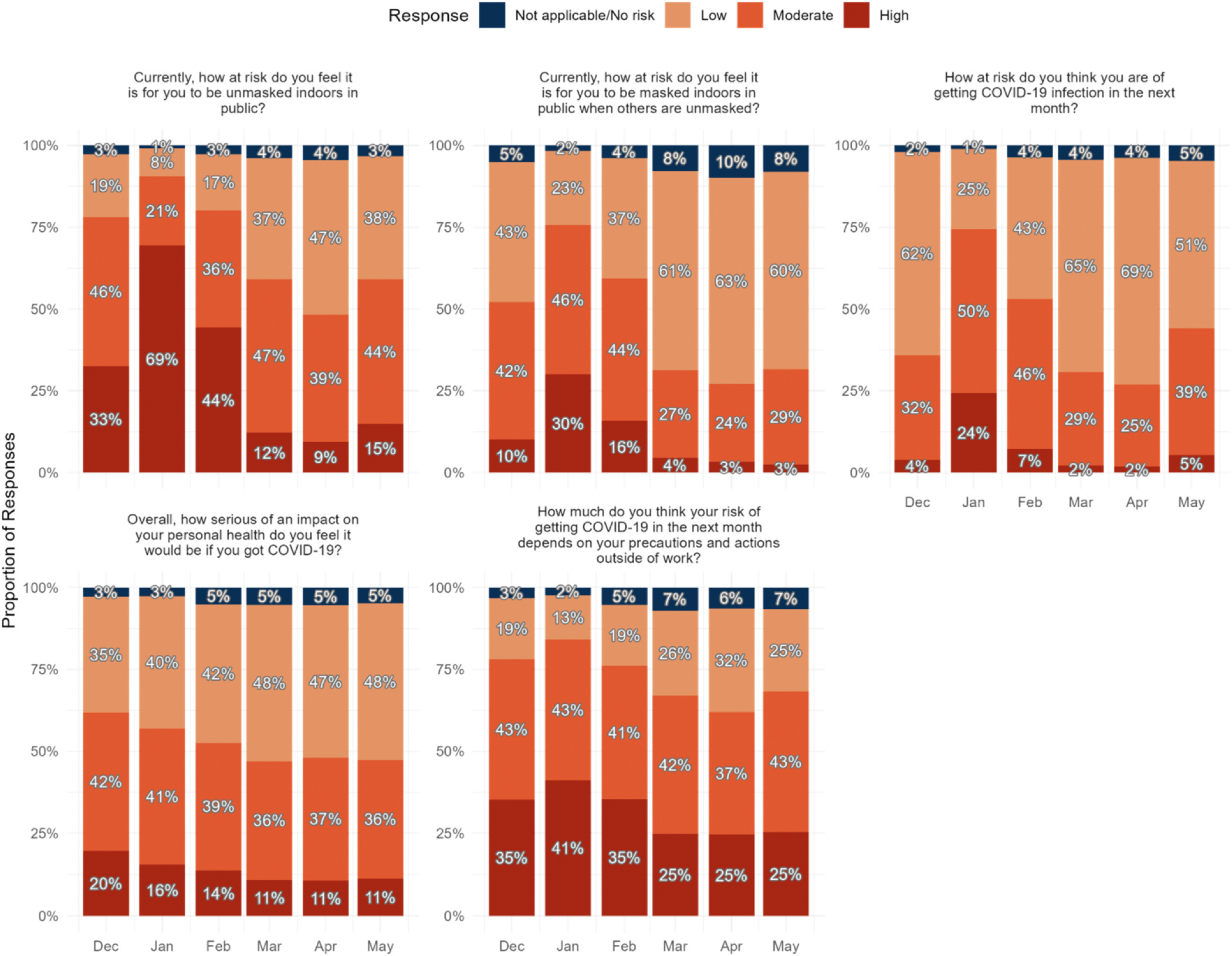
Proportion of responses for risk perception questions over the December 2021 to May 2022 study period. Questions assessing impact on personal health and how much risk depends on precautions and actions outside of work originally had 5 response options, with the last 2 condensed here for simplicity (originally “No Impact”/”Not dependent” and “Not applicable”).

**Fig. 3.**
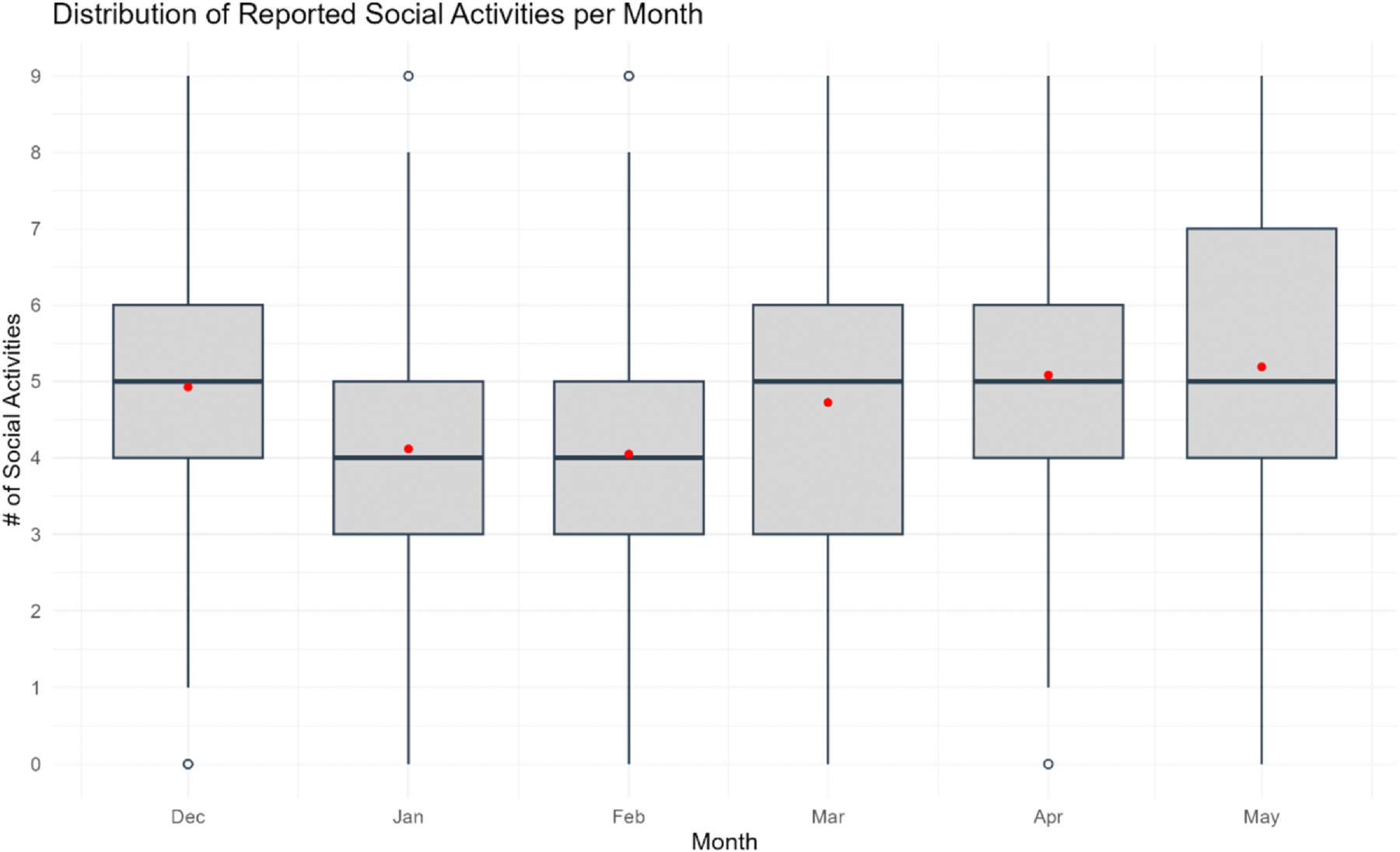
Box plot of the count of social activities reported in each survey month. Lines represent medians, while red dots represent means.

The proportion of survey responses indicating high-risk perception varied over time, with the highest risk perceptions in January and February (Fig. 2). Social activity followed a similar, though more subtle, pattern (Fig. 3). Of all risk perception questions, high risk perception was reported most frequently for the risk of being unmasked indoors in public during January and February. All risk perception questions showed low (r = 0.25) to moderate (r = 0.64) correlation with each other, while all risk perception questions were inversely correlated with social activity count (Supplementary Figs. S3, S4).

### Model results

#### Convergence checks

We utilized Gelman-Rubin $\overset{ˆ}{R}$ statistics to test for convergence, and all were below 1.01. Trace plots can be viewed in the Supplementary Material (Fig. S2).

#### Main results

Our model results show significant associations between all risk perception variables and high COVID-19 incidence, compared to low incidence levels (Table 2, Supplementary Table S4). Number of reported social activities and perceived impact on personal health were inversely associated. On average, individuals reported 0.68 (95% CrI: −0.88, −0.47) fewer social activities during high incidence levels than during low incidence levels. Perceived risk of infection in the next month and risk while unmasked indoors in public had the largest associations of the risk perception variables with high incidence levels, with an average of 0.60 (95% CrI: 0.51, 0.69) and 0.62 (95% CrI: 0.52,0.71) higher scores during periods of high incidence compared to low incidence. Perceived risk while unmasked indoors in public showed significant associations with all levels of incidence compared to low levels.

Residual outcome correlations from the model were generally lower than the raw correlations, as expected (Supplementary Fig. S5). The 2 masking outcomes had the highest residual correlation (R^2^ = 0.49), followed by risk while unmasked indoors in public and risk of infection in the next month (R^2^ = 0.40). This suggests that the correlation between these variables is not entirely explained by the variables of the model.

## DISCUSSION

Our analysis of local COVID-19 incidence, risk perception, and reported social activity among HCPs found local incidence levels to be significantly associated with multiple risk perception measures. Limiting social activity and perceived impact on personal health were associated with local incidence levels. These associations generally became stronger as incidence levels increased. These results suggest that HCP responded to the changing levels of disease around them with both increased perception of risk and reduced social activities.

Other studies have shown several measures of local disease risk, such as death rates or local incidence rates, to be associated with risk perception^20,21^ and reduced social activity^21^ in the general population. COVID-19 risk perception has also been shown to be generally decreasing over time.^22,23^ However, some research conducted during the emergence of Omicron showed an increase in perceived likelihood of infection, similar to our findings.^22,24^ These effects may be magnified in our sample, as HCP may have a different perception of risk compared to the general population, perhaps because of their intimate exposure to severe illness and death.^5^ Similar to our results, higher risk perception has generally been associated with better adherence to preventative behaviors.^19,20,25^ However, different types of risk perception, such as severity of illness, may have a greater impact on preventative behaviors.^22,26^

We found a weak inverse relationship between perceptions of serious risk to personal health and high local incidence in our adjusted model. We expected that perceptions of the impact on personal health would not be associated with local incidence, as previous research has shown perceived severity to be a distinct concept from perceived susceptibility.^27^ There is evidence that the Omicron strain of SARS-CoV-2, which was prominent during the study, was associated with less severe illness but increased contagiousness.^28^ At least one other study also found an inverse relationship between daily registered infections and perceived severity.^22^ It is possible that, as cases increased and individuals observed more people with mild (rather than severe) illness, their perceptions of how COVID-19 would impact them personally changed to fit a milder illness.

Among all the risk perception questions in this survey, HCP reported feeling at highest risk when unmasked indoors in public. In contrast, their perceived risk was much lower when respondents were asked about themselves being masked while others were not. This suggests that HCP placed high value on the individual protective effect of masks. Alternatively, being unmasked yourself may be seen as riskier because you have control over it, whereas you don’t have control over the masking of others. Research shows that masks are effective at reducing transmission^29–32^ through source control and as individual protection. However, in situations where only one person is masked, they are more effective at source control.^33^ If the difference in responses related to masking is due to a lack of knowledge, there may be educational opportunities for HCPs to improve their knowledge of mask effectiveness. If the differences stem from a sense of control over risk, this could be an area to emphasize in risk messaging to encourage preventative measures during periods of high incidence. County-level mask mandates were in place during part of the study, which also could have influenced respondents’ risk perceptions. While prior research has examined how HCP risk perception influences masking and PPE adherence,^34^ we are not aware of studies specifically investigating how medical mask mandates affect HCP risk perception. Some studies suggest that HCP were generally supportive of universal masking policies, though this support appeared stronger earlier in the pandemic than in later phases.^35^

This study has several limitations. As an observational study, this study cannot directly establish causation; it can only provide associations and supporting evidence. It also utilizes self-reported survey data, which can be subject to biases, such as social desirability or selection bias. Because participants were not compensated, their participation likely reflected a degree of self-motivation. As a result, individuals more engaged with public health or supportive of COVID-19 prevention measures may have been more inclined to respond. Additionally, social desirability bias may have influenced participants’ self-reports of risky or preventive behaviors. However, prior research indicates that self-reported social distancing during COVID-19 aligns with actual social distancing observed through mobility data.^36^ As this study includes only HCP, it may not be generalizable to the broader population. However, the longitudinal design strengthens the analysis and addresses a gap that is often overlooked in other risk perception studies. Dropout occurred within our study, but we have used weighting to minimize its effect. Although we assessed risk perception in the same individuals over time, the analysis does not adjust for variability in how likely individuals are to change their responses. Finally, it is possible that other variables we have not measured may impact the relationships we observed here. For example, trust in government^37,38^ and trust in health experts^37^ have been associated with risk perception and preventative measures in cross-sectional research. Future research on risk perception and behavior could incorporate these variables and observe their changes over time.

## CONCLUSIONS

In this study, we found local COVID-19 incidence levels to be associated with risk perception and reported social activity among HCP who were experiencing the impact of the pandemic up close. These results show that HCP responded to the changing risk around them. We also found HCP placed high value on individual masking. This can inform tailored messaging and support for HCP across incidence levels. Our response to future pandemics can be guided by the knowledge that as local risk changes, HCP risk perceptions and behaviors change with them.

## Supplementary Material

1

## Figures and Tables

**Table 1:** Demographic characteristics of the study participants

Characteristic	N = 1,590*

Age	
18-24	151 (9.5%)
25-34	479 (30%)
35-44	457 (29%)
45-54	292 (18%)
55-64	170 (11%)
65+	40 (2.5%)
Unknown	1
Gender	
Male	327 (21%)
Female	1,249 (79%)
Unknown	14
Occupational role	
Nurse	523 (33%)
Nurse assistants (CNA, HCA, MA, or EMT)	297 (19%)
Physician/APC	321 (20%)
Other	449 (28%)
Work location	
Outpatient ambulatory	561 (35%)
Inpatient acute care	259 (16%)
Critical care	250 (16%)
Inpatient psychiatry	52 (3.3%)
Others	468 (29%)
General health status	
Excellent	402 (25%)
Very good	769 (48%)
Good or below	415 (26%)
Unknown	4
Any comorbidity	363 (23%)
Household status	
Living alone	164 (10%)
Living with others, no school-age children	953 (60%)
Living with others, 1+ school-age children	473 (30%)

* n (%).

**Table 2:** Coefficients shown from a Bayesian multivariate Gaussian model assessing the association between 5 risk perception outcomes and social activity with local COVID-19 incidence levels

Covariate	Risk next month (susceptibility)	Unmasked risk (self-efficacy and susceptibility)	Masked risk (susceptibility)	Impact (severity)	Precautions (self-efficacy)	Social activity

*Incidence levels*						
Moderate	0.00 (−0.08, 0.08)	**0.11 (0.03, 0.20)**	−0.06 (−0.15, 0.03)	−0.02 (−0.12, 0.08)	0.04 (−0.05, 0.13)	−0.04 (−0.22, 0.14)
Substantial	**0.12 (0.03, 0.21)**	**0.22 (0.12, 0.32)**	0.01 (−0.10, 0.11)	−0.01 (−0.13, 0.11)	0.10 (−0.00, 0.21)	**−0.32 (−0.53, −0.10)**
High	**0.60 (0.51, 0.69)**	**0.62 (0.52, 0.71)**	**0.41 (0.31, 0.50)**	**−0.12 (−0.23, −0.01)**	**0.13 (0.03, 0.23)**	**−0.68 (−0.88, −0.47)**

NOTE. Reported in the table as coefficient (95% Credible intervals).

Significant 95% credible intervals are shown in bold.

## APPENDIX A. SUPPLEMENTARY DATA

Supplementary data related to this article can be found at doi:10.1016/j.ajic.2025.10.013.
