# Supplementary material for: Impact of local COVID-19 incidence on health care personnel risk perception and social activity
Source: Am J Infect Control. Author manuscript; Available in PMC 2026 Jul 20. (PMC13384357; doi:10.1016/j.ajic.2025.10.013)
Supplement: 1 [file NIHMS2186262-supplement-1.docx]

Supplement

Impact of Local COVID-19 Incidence on Healthcare Personnel Risk Perception and Social Activity

Table of Contents

[Supplement 1](#_Toc200389765)

[Figures 2](#_Toc200389766)

[Figure S1 Covariate Balance Plot 2](#_Toc200389767)

[Figure S2 Bayesian Joint Model Trace Plots 3](#_Toc200389768)

[Figure S3 Social Activity and Risk Perception Correlation Overall 4](#_Toc200389769)

[Figure S4 Social Activity and Risk Perception Correlation by Month 5](#_Toc200389770)

[Figure S5 Residual Correlation of Outcomes 6](#_Toc200389771)

[Tables 7](#_Toc200389772)

[Table S1 Risk Perception Questions 7](#_Toc200389773)

[Table S2 Social Activity List 8](#_Toc200389774)

[Table S3 Collapsed Variables 9](#_Toc200389775)

[Table S4 Full Model With Covariates 11](#_Toc200389776)

## Figures

### Figure S1 Covariate Balance Plot

Balance of covariates used in dropout weighting. Covariate balancing propensity score methods were used to create the weights, with a 0.1 standardized mean difference threshold.


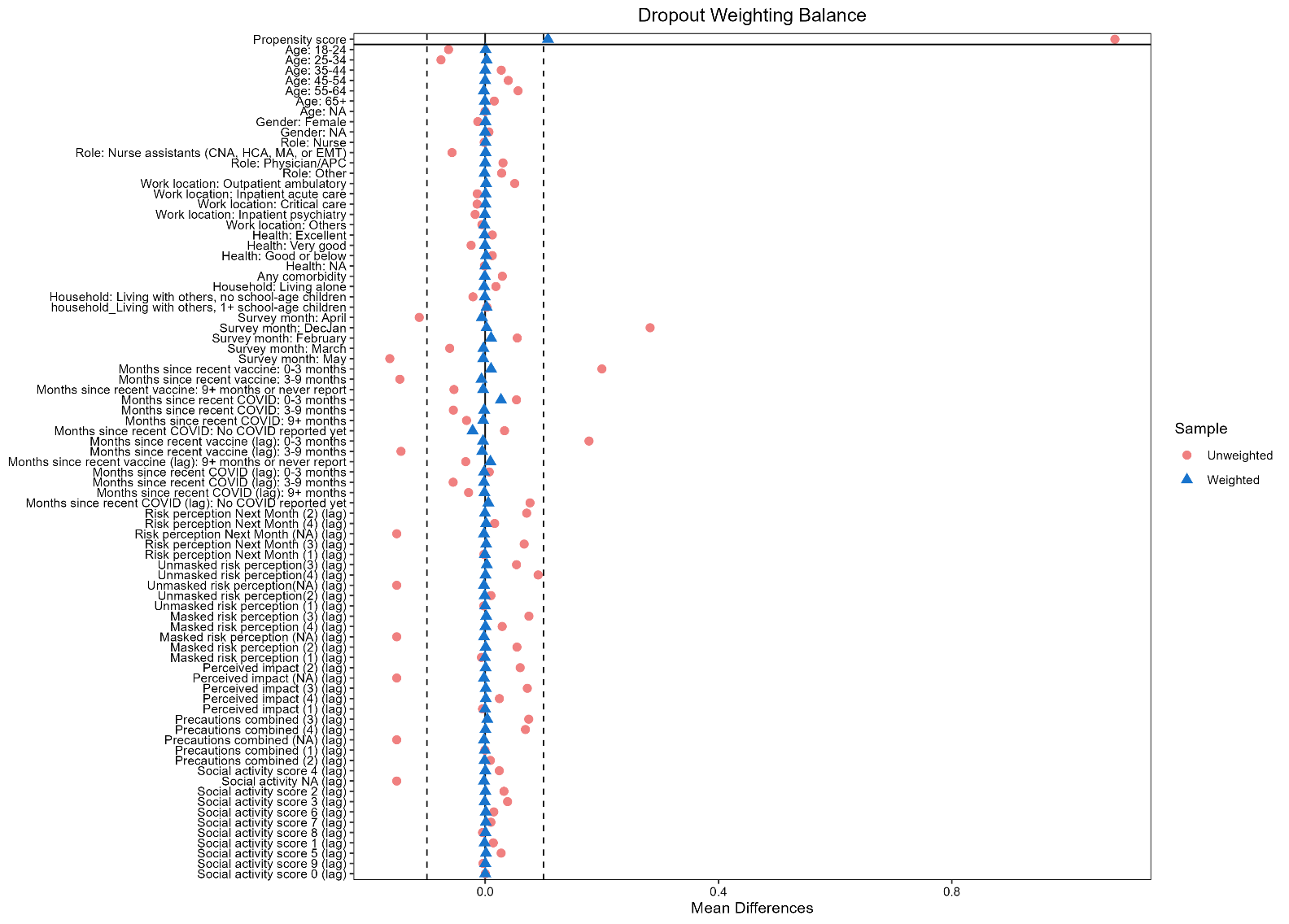


### Figure S2 Bayesian Joint Model Trace Plots

Trace plots showing the convergence for the last half of the iterations for the model. Only the main exposure variable—incidence levels—is included.


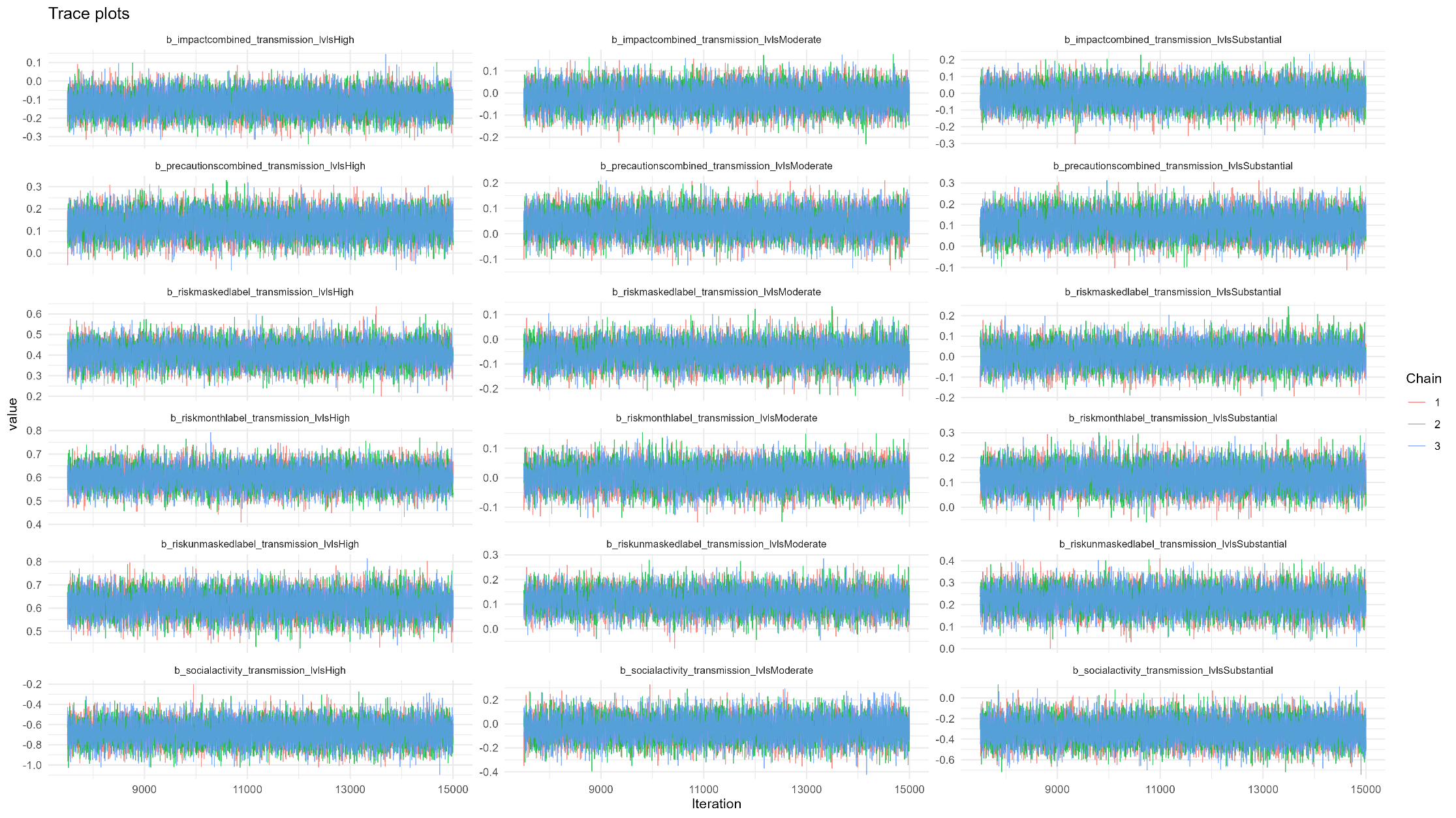


### Figure S3 Social Activity and Risk Perception Correlation Overall

Pearson Correlation of all outcome variables at all time points.


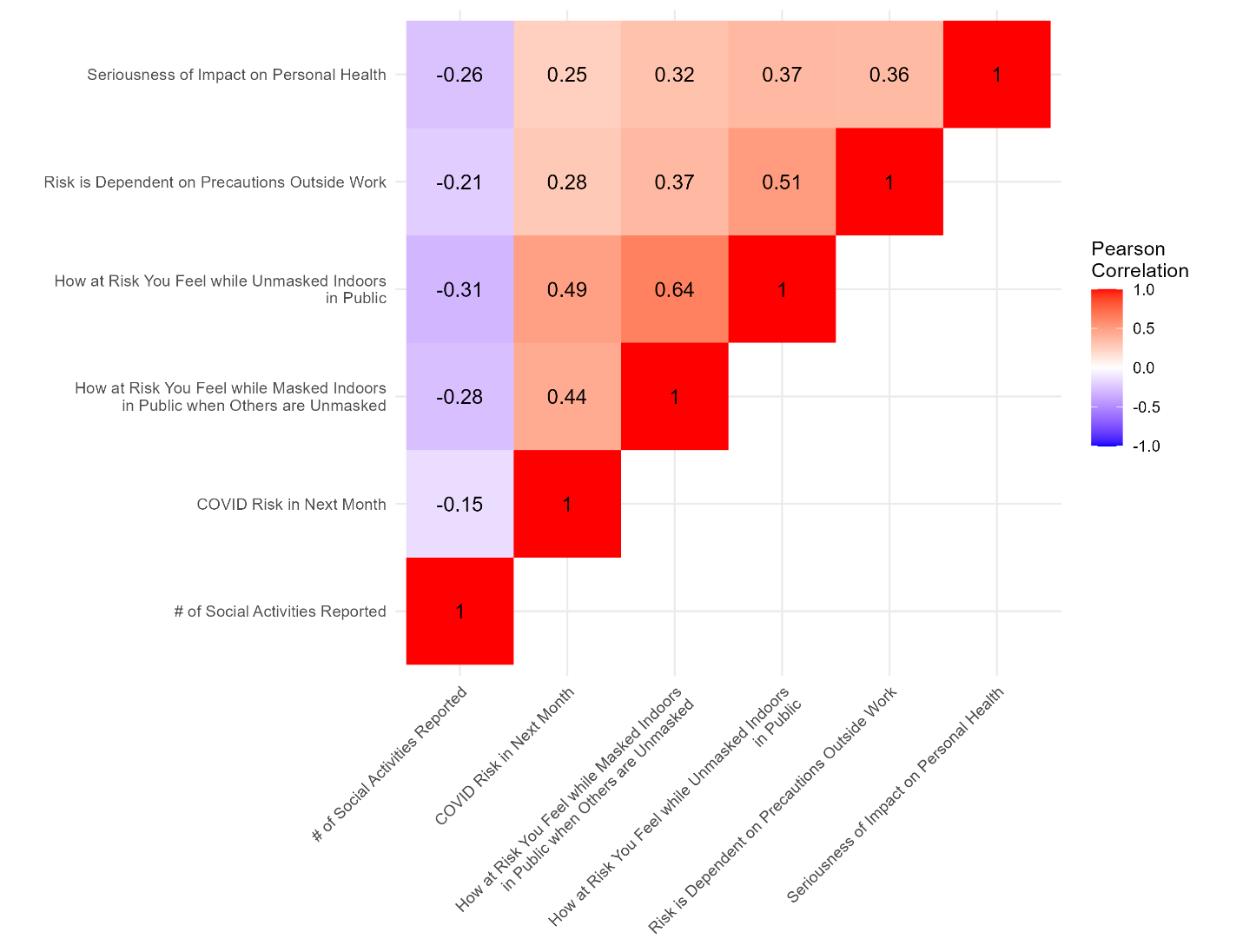


### Figure S4 Social Activity and Risk Perception Correlation by Month

Pearson Correlation of all outcome variables at each survey month.


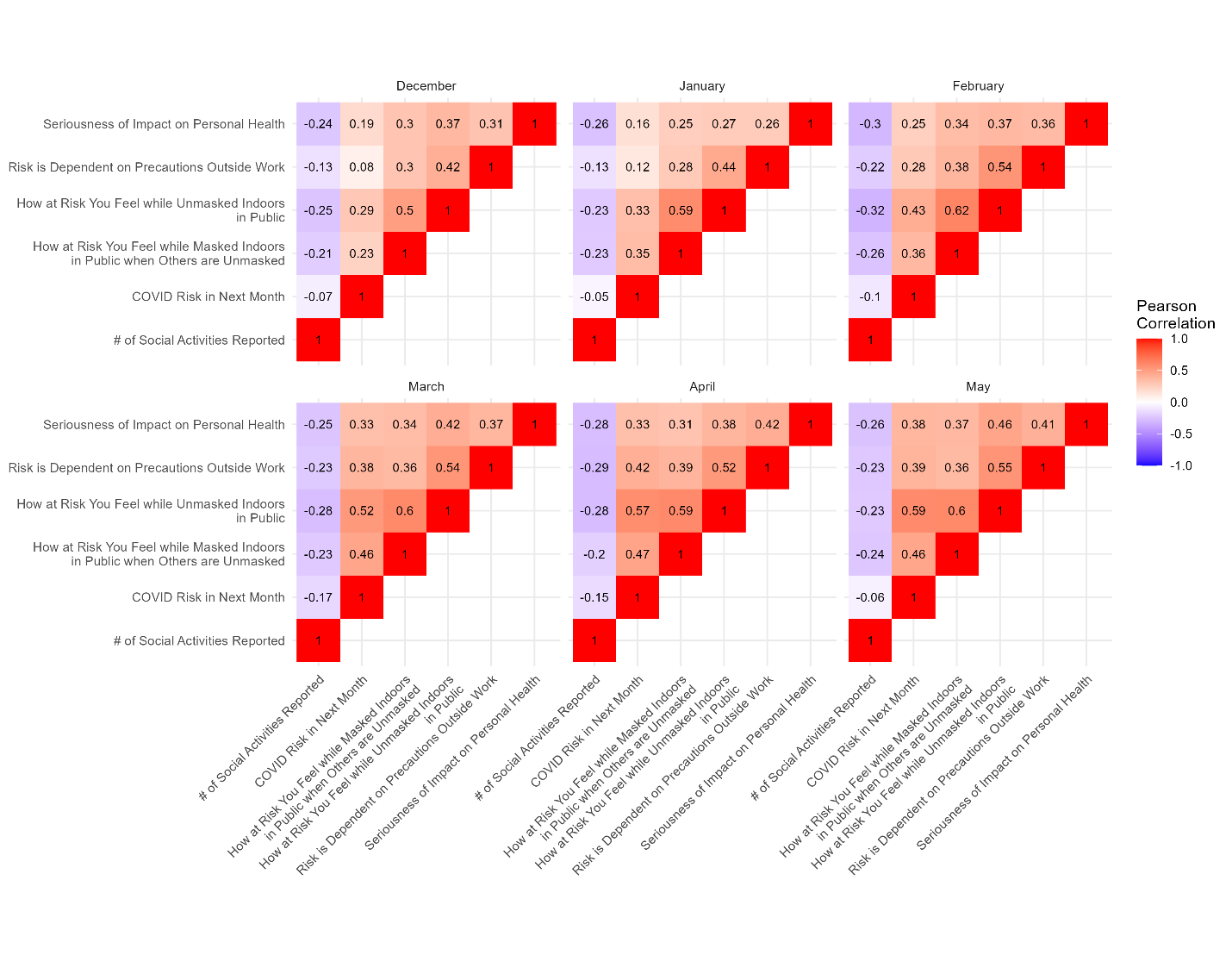


### Figure S5 Residual Correlation of Outcomes

Residual correlation of all outcomes after modeling with a multivariate Bayesian joint model.


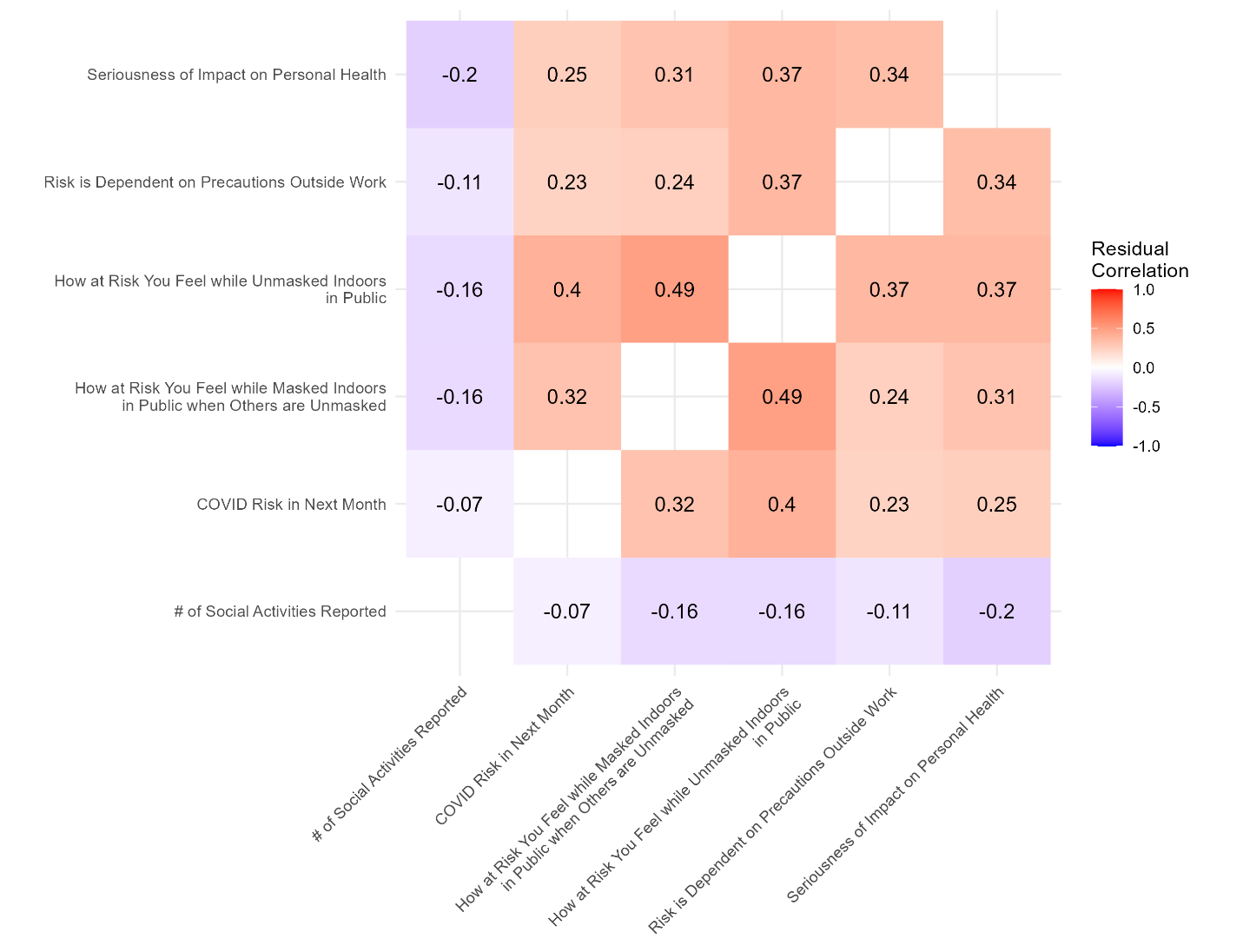


## Tables

### Table S1 Risk Perception Questions

| Question Text | Response Options |
| --- | --- |
| *How at risk do you think you are of getting COVID-19 infection in the next month? Would you say that you are at:* | 4-point Likert: High risk, Moderate risk, Low risk, No risk |
| *Currently, how at risk do you feel it is for you to be unmasked indoors in public?* | 4-point Likert: High risk, Moderate risk, Low risk, No risk |
| *Currently, how at risk do you feel it is for you to be masked indoors in public when others are unmasked?* | 4-point Likert: High risk, Moderate risk, Low risk, No risk |
| *Overall, how serious of an impact on your personal health do you feel it would be if you got COVID-19?* | 5-point Likert: Significant impact, Moderate impact, Small impact, No impact, Not applicable, I am not at risk of getting COVID-19 |
| *How much do you think your risk of getting COVID-19 in the next month depends on your precautions and actions outside of work? Is your risk* | 5-point Likert: Highly dependent upon my precautions/actions, Moderately dependent upon my precautions/actions, Slightly dependent upon my precautions/actions, Not at all dependent upon my precautions/actions, Not applicable, I am not at risk of getting COVID-19 |

### Table S2 Social Activity List

| Question Text | Response Options |
| --- | --- |
| *In the past month, did you do any of the following?* | Checkbox;   - Socialized at a home indoors with non-household members - Socialized outdoors with non-household members - Socialized or attended an indoor event in public (i.e., concert/movies/sporting) - Socialized or attended an outdoor event in public (i.e., concert/movies/sporting) - Attended in-person religious services - Gone to a store (i.e., grocery, retail, etc.) - Gone to a gym or fitness center - Eaten indoors at a restaurant - Traveled on an airplane - None of the above |

### Table S3 Collapsed Variables

| Collapsed Variables | | |
| --- | --- | --- |
| Name | **Original Options** | **Condensed** |
| Work Role | Physician | Nurse |
|  | APC (e.g., PA, APRN, etc.) | Nurse assistants (CNA, HCA, MA, or EMT) |
|  | Nurse | Physician/APC |
|  | CNA, HCA, or MA | Other, including physical therapist, respiratory therapist, pharmacist, patient relations specialist, technologist, and the “Other (please specify)” category |
|  | EMT |  |
|  | Physical Therapist |  |
|  | Respiratory Therapist |  |
|  | EVS or Housekeeper |  |
|  | Pharmacist |  |
|  | Patient Relations Specialist |  |
|  | Transport |  |
|  | Technologist |  |
|  | Other (please specify) |  |
| Work Location | University Hospital Critical Care Unit | Outpatient ambulatory, including ambulatory clinic and urgent care |
|  | University Hospital Acute Care Unit | Inpatient acute care, including acute care and inpatient rehab unit within the academic healthcare system |
|  | Huntsman Cancer Hospital Critical Care Unit | Critical care, including critical care units and emergency department |
|  | Huntsman Cancer Hospital Cancer Acute Care Unit | Inpatient psychiatry |
|  | HMHI Inpatient Psychiatric Unit 6 NRH Inpatient Rehab Unit | Others, including perioperative or operating room (OR), and “Other (please specify). |
|  | Emergency Department 8 Perioperative/OR |  |
|  | Ambulatory Clinic |  |
|  | Urgent Care |  |
|  | Non-surgical Procedural or Diagnostic Areas |  |
|  | Other (please specify) |  |
| Any Comorbidity | Diabetes | Binary Indicator for whether the HCP had any diabetes, hypertension or high blood pressure, cardiovascular disease or heart disease, pulmonary disease, any condition requiring immunosuppressive therapy, immunocompromised condition, autoimmune disease, and/or kidney disease |
|  | Hypertension or High Blood Pressure |  |
|  | Cardiovascular Disease or Heart Disease |  |
|  | Pulmonary Disease (for example, Asthma or Cystic Fibrosis) |  |
|  | Any Condition Requiring Immunosuppressive Therapy |  |
|  | Immunocompromised Condition |  |
|  | Autoimmune Disease |  |
|  | Kidney Disease |  |
|  | Solid Organ Transplant (within the last 24 months) |  |
|  | Cancer (current, in treatment, or diagnosed within the last 12 months) |  |
|  | Prefer Not to Answer |  |
|  | None of the Above |  |
| Household Status | How many people live in your household (1 to 6+) | Living alone |
|  | Of these people that live in your household, how many are children under age 12? (0 to 4 or more) | Living with others, no school-age children |
|  | How many of the children under age 12 in your household attend school or day care outside of the home? (0 to 4 or more) | Living with others, 1+ school-age children |

### Table S4 Full Model With Covariates

Full Table with all covariates

Coefficients shown from a Bayesian multivariate Gaussian model assessing the association between five risk perception outcomes and social activity with local COVID-19 incidence levels. All covariates shown. 95% Credible intervals are shown in parentheses.

| Bayesian Multivariate Model Results | | | | | | |
| --- | --- | --- | --- | --- | --- | --- |
| Coefficients (95% CrI) for Gaussian Model | | | | | | |
| Covariate | Risk (Month) | Unmasked Risk | Masked Risk | Impact | Precautions | Social Activity |
| ***Model Intercept*** | | | | | | |
| Intercept | **1.88 (1.74, 2.03)** | **2.25 (2.08, 2.41)** | **2.09 (1.93, 2.25)** | **1.91 (1.78, 2.04)** | **2.32 (2.11, 2.53)** | **6.56 (6.06, 7.05)** |
| ***Incidence Levels*** | | | | | | |
| Moderate | 0.00 (-0.08, 0.08) | **0.11 (0.03, 0.20)** | -0.06 (-0.15, 0.03) | -0.02 (-0.12, 0.08) | 0.04 (-0.05, 0.13) | -0.04 (-0.22, 0.14) |
| Substantial | **0.12 (0.03, 0.21)** | **0.22 (0.12, 0.32)** | 0.01 (-0.10, 0.11) | -0.01 (-0.13, 0.11) | 0.10 (-0.00, 0.21) | **-0.32 (-0.53, -0.10)** |
| High | **0.60 (0.51, 0.69)** | **0.62 (0.52, 0.71)** | **0.41 (0.31, 0.50)** | **-0.12 (-0.23, -0.01)** | **0.13 (0.03, 0.23)** | **-0.68 (-0.88, -0.47)** |
| ***Sex*** | | | | | | |
| Female | 0.02 (-0.04, 0.07) | **0.18 (0.12, 0.25)** | 0.06 (-0.00, 0.11) | **0.17 (0.13, 0.21)** | **0.13 (0.05, 0.21)** | **-0.29 (-0.48, -0.10)** |
| ***Age Group*** | | | | | | |
| 25 to 34 | **-0.11 (-0.19, -0.02)** | -0.04 (-0.14, 0.06) | -0.01 (-0.10, 0.09) | 0.04 (-0.03, 0.10) | 0.12 (-0.01, 0.24) | **-0.70 (-0.99, -0.39)** |
| 35 to 44 | -0.09 (-0.19, 0.00) | -0.02 (-0.13, 0.09) | -0.00 (-0.11, 0.10) | **0.10 (0.04, 0.17)** | **0.17 (0.03, 0.30)** | **-1.03 (-1.35, -0.69)** |
| 45 to 54 | **-0.19 (-0.28, -0.10)** | -0.06 (-0.17, 0.05) | -0.02 (-0.13, 0.08) | **0.12 (0.05, 0.19)** | **0.18 (0.04, 0.32)** | **-1.09 (-1.42, -0.75)** |
| 55 to 64 | **-0.27 (-0.37, -0.16)** | -0.01 (-0.13, 0.12) | -0.00 (-0.12, 0.12) | **0.32 (0.25, 0.40)** | **0.21 (0.06, 0.37)** | **-1.28 (-1.64, -0.91)** |
| 65+ | **-0.26 (-0.41, -0.10)** | 0.11 (-0.07, 0.29) | **0.17 (0.00, 0.35)** | **0.39 (0.27, 0.50)** | **0.39 (0.16, 0.62)** | **-1.21 (-1.77, -0.63)** |
| ***Role*** | | | | | | |
| Nurse assistants, CNA, HCA, MA, or EMT | -0.03 (-0.09, 0.04) | 0.00 (-0.08, 0.08) | 0.00 (-0.07, 0.08) | **0.06 (0.02, 0.11)** | -0.01 (-0.11, 0.09) | **-0.60 (-0.83, -0.35)** |
| Physician or APC | **0.12 (0.05, 0.18)** | **0.21 (0.14, 0.29)** | **0.10 (0.03, 0.18)** | 0.02 (-0.03, 0.06) | **0.13 (0.04, 0.23)** | -0.15 (-0.37, 0.08) |
| Other | -0.00 (-0.06, 0.05) | **0.10 (0.03, 0.16)** | -0.01 (-0.07, 0.05) | **0.05 (0.01, 0.09)** | 0.07 (-0.02, 0.15) | -0.17 (-0.37, 0.03) |
| ***Comorbidities*** | | | | | | |
| One or more | -0.00 (-0.05, 0.05) | 0.03 (-0.03, 0.09) | -0.01 (-0.07, 0.05) | **0.20 (0.16, 0.24)** | 0.01 (-0.07, 0.09) | **-0.22 (-0.41, -0.02)** |
| ***Health Status*** | | | | | | |
| Very good | **0.12 (0.07, 0.17)** | 0.04 (-0.02, 0.11) | 0.05 (-0.01, 0.11) | **0.13 (0.09, 0.17)** | 0.01 (-0.07, 0.09) | **-0.26 (-0.45, -0.07)** |
| Good or below | **0.21 (0.15, 0.28)** | **0.17 (0.09, 0.24)** | **0.14 (0.07, 0.21)** | **0.47 (0.43, 0.52)** | 0.08 (-0.01, 0.18) | **-0.79 (-1.02, -0.56)** |
| ***Months Since Recent Vaccine*** | | | | | | |
| Vaccine 3 to 9 months | **0.11 (0.07, 0.15)** | -0.01 (-0.05, 0.03) | -0.01 (-0.05, 0.04) | 0.05 (-0.00, 0.10) | 0.03 (-0.02, 0.08) | **-0.09 (-0.19, -0.00)** |
| Vaccine 9 + months or never report | -0.02 (-0.07, 0.04) | **-0.21 (-0.27, -0.15)** | **-0.17 (-0.24, -0.12)** | **-0.07 (-0.12, -0.01)** | **-0.25 (-0.32, -0.18)** | **0.24 (0.09, 0.39)** |
| ***Months Since Recent COVID*** | | | | | | |
| COVID 3+ months | **0.31 (0.26, 0.36)** | 0.00 (-0.05, 0.06) | **0.07 (0.01, 0.13)** | **0.10 (0.04, 0.16)** | **0.16 (0.09, 0.22)** | **0.30 (0.17, 0.43)** |
| No COVID reported yet | **0.44 (0.39, 0.49)** | **0.20 (0.15, 0.26)** | **0.16 (0.10, 0.21)** | **0.18 (0.13, 0.24)** | **0.32 (0.26, 0.39)** | **0.15 (0.03, 0.29)** |
| ***Work Location*** | | | | | | |
| Inpatient Acute Care | 0.00 (-0.06, 0.07) | 0.03 (-0.05, 0.11) | 0.04 (-0.03, 0.11) | 0.03 (-0.02, 0.08) | -0.02 (-0.11, 0.08) | 0.03 (-0.21, 0.26) |
| Critical Care | **0.12 (0.06, 0.19)** | 0.06 (-0.02, 0.14) | 0.02 (-0.06, 0.09) | -0.02 (-0.07, 0.02) | -0.02 (-0.12, 0.08) | 0.13 (-0.10, 0.37) |
| Inpatient Psychiatry | 0.01 (-0.11, 0.13) | 0.11 (-0.04, 0.25) | 0.07 (-0.07, 0.21) | 0.07 (-0.02, 0.16) | 0.08 (-0.10, 0.26) | 0.21 (-0.22, 0.65) |
| Other Location | **-0.06 (-0.11, -0.00)** | **-0.10 (-0.17, -0.04)** | -0.06 (-0.12, 0.00) | 0.02 (-0.02, 0.06) | -0.03 (-0.11, 0.05) | 0.17 (-0.02, 0.37) |
| ***Household Status*** | | | | | | |
| Living with Others No School-age Children | **-0.10 (-0.17, -0.03)** | -0.07 (-0.15, 0.02) | -0.07 (-0.14, 0.01) | -0.04 (-0.09, 0.01) | -0.07 (-0.17, 0.04) | 0.11 (-0.14, 0.37) |
| Living with Others 1+ School-age Children | **-0.08 (-0.16, -0.00)** | **-0.16 (-0.25, -0.07)** | **-0.10 (-0.19, -0.01)** | **-0.11 (-0.17, -0.05)** | **-0.23 (-0.34, -0.11)** | 0.14 (-0.14, 0.43) |
| ***Survey Month*** | | | | | | |
| December/January | **0.15 (0.07, 0.23)** | **0.42 (0.33, 0.51)** | **0.38 (0.29, 0.47)** | **0.27 (0.17, 0.38)** | **0.28 (0.18, 0.37)** | **-0.28 (-0.47, -0.08)** |
| February | **0.29 (0.21, 0.37)** | **0.54 (0.45, 0.63)** | **0.51 (0.42, 0.61)** | 0.09 (-0.02, 0.20) | **0.20 (0.11, 0.30)** | **-0.84 (-1.03, -0.65)** |
| March | **0.06 (0.02, 0.10)** | **0.16 (0.12, 0.20)** | **0.08 (0.04, 0.12)** | -0.01 (-0.07, 0.04) | **0.06 (0.02, 0.11)** | **-0.33 (-0.41, -0.24)** |
| May | **0.19 (0.12, 0.27)** | 0.06 (-0.02, 0.15) | 0.09 (-0.00, 0.18) | 0.01 (-0.09, 0.11) | 0.01 (-0.08, 0.10) | 0.16 (-0.02, 0.35) |
